# Respiratory Syncytial Virus Prevention: A New Era of Vaccines

**DOI:** 10.7759/cureus.45012

**Published:** 2023-09-11

**Authors:** Calvin Yee Fen Lee, Salman J Khan, Fnu Vishal, Sadaf Alam, Syeda Fatima Murtaza

**Affiliations:** 1 Internal Medicine, Jiangsu University, Zhenjiang, CHN; 2 Public Health, University of Massachusetts, Amherst, USA; 3 Hematology and Oncology, Mayo Clinic, Jacksonville, USA; 4 Public Health, Johns Hopkins Bloomberg School of Public Health, Baltimore, USA; 5 Infectious Diseases, Rochester General Hospital, Rochester, USA; 6 Neurology, Mir Neurology, Hagerstown, USA; 7 Medicine, Mayo Clinic, Jacksonville, USA; 8 Medicine, Allama Iqbal Medical College, Lahore, PAK

**Keywords:** lower respiratory tract infection, rsv vaccine, rsvpref3 oa, nirsevimab, respiratory syncytial virus (rsv)

## Abstract

Respiratory syncytial virus (RSV) is a pathogen that primarily affects the respiratory system, leading to upper and lower respiratory tract infections. Children, individuals aged 60 and above, and individuals with impaired immune systems are more susceptible to developing RSV lower respiratory tract infections (LRTIs), which can result in fatalities in some instances. Symptoms of LRTI include shortness of breath, wheezing, pneumonia, and bronchiolitis. Current management of RSV-LRTI includes conservative and symptomatic treatment. The Food and Drug Administration (FDA) recently approved two vaccines that effectively prevent acute and severe RSV-LRTI requiring hospitalizations. Nirsevimab (Beyfortus) is approved for infants born at 35 weeks of gestation and above. At the same time, RSVPreF3 OA (Arexvy) is recommended for adults aged 60 and older. Both vaccines are effective against the two major strains of RSV and require single doses to induce immunity. In this article, we will discuss the mechanism of action, effectiveness, and side effects of these novel vaccines and their possible impact.

## Editorial

Respiratory syncytial virus (RSV) is a non-segmented, negative-sense, single-stranded RNA virus. RSV A and RSV B are the major variants causing lower respiratory tract infections (LRTI) in children, the elderly, and other immunocompromised individuals. Around 175,000 children under five years of age are hospitalized annually, and about 14,000 older adults die annually in the United States alone [1]. RSV causes 3.4 million hospitalizations and 95,000-150,000 deaths worldwide annually [2]. Symptoms of LRTI include pneumonia, wheezing, inflammation of the bronchioles, and shortness of breath, which may sometimes be fatal. In the pediatric and adult populations, RSV treatment is limited to conservative and symptomatic management. Recently, the FDA approved nirsevimab (Beyfortus) and RSVPreF3 OA (Arexvy) to prevent RSV-LRTI in infants and adults aged 60 years and older, respectively. Studies have shown that these vaccines are significantly effective against RSV infection and its associated complications, including hospitalizations and deaths.

Nirsevimab, approved by the FDA in July 2023, is a recombinant human IgG1κ monoclonal antibody designed to target the pre-F protein in RSV. The virus adheres to cell membranes by changing the pre-fusion (pre-F) structure to a stable post-fusion (post-F) structure [2]. The F-protein is highly conserved among RSV strains, thus providing a target broadly covering both RSV A and B. Nirsevimab inhibits the required conformational changes in pre-F, which prevents the adherence of the virus to cell membranes. A phase 3 trial, which included 1,490 infants born at term and greater than or equal to 35 weeks (994 nirsevimab and 496 placebo), showed that 1.2% of infants developed RSV-LRTI in the nirsevimab group compared to 5.0% of infants in the placebo group, showing a relative risk reduction (RRR) of around 75% compared to placebo [3]. Another randomized, double-blind, multicenter trial provided more evidence supporting the utilization of nirsevimab in children aged up to 24 months who continue to be at risk for severe RSV sickness during their second RSV season. The study included a total of 925 participants, comprising preterm newborns as well as infants diagnosed with chronic lung disease of prematurity or congenital heart disease. During a 150-day observation period after administration, the nirsevimab group showed an RRR of 79.5% in preventing RSV-LRTI compared to the placebo. Additionally, the nirsevimab group exhibited an RRR of 43.8% for hospital admissions related to respiratory illnesses of any cause compared to the placebo [4].

RSVPreF3 OA is another vaccine approved for adults aged 60 years and older by the FDA in 2023. It also targets the pre-F protein. It uses a recombinant RSV glycoprotein F stabilized in pre-fusion conformation (RSVPreF3) as the antigen component. It works by inducing an immune response against RSVPreF3, which confers immunity against RSV subtypes. A phase 3 trial involving 24,966 participants aged 60 and older (12467 RSVPreF3 OA and 2499 placebo) revealed a notable decrease in the incidence of RSV-associated lower respiratory tract disease (LRTD) by 82.6% in the RSVPreF3 OA group compared to the placebo group. The trial also showed a significant reduction of 94.1% in the likelihood of having severe RSV-associated LRTD in the RSVPreF3 OA group compared to the placebo group [5].

The prevailing adverse reactions documented among persons who were administered these vaccines include headache, malaise, weariness, and arthralgias [3]. The majority of adverse events were temporary and exhibited mild to moderate severity. The rates of severe adverse events and possible immune-mediated illnesses were comparable across the two groups [5].

These newly approved vaccines can decrease morbidity and mortality in children and older people. Another benefit is the single dosing required for both patient populations, which may increase acceptance among the patient population. The safety and efficacy of vaccines have been demonstrated irrespective of underlying diseases and the specific subtype of respiratory syncytial virus (RSV). Including these vaccines in the routine vaccination guidelines may reduce RSV-related hospitalizations and deaths, leading to better disease outcomes.
